# Previous Exercise on a Water Treadmill at Different Depths Affects the Accelerometric Pattern Recorded on a Track in Horses

**DOI:** 10.3390/ani12223086

**Published:** 2022-11-09

**Authors:** Aritz Saitua, Cristina Castejón-Riber, Francisco Requena, David Argüelles, Natalie Calle-González, Antonia Sánchez de Medina, Ana Muñoz

**Affiliations:** 1 Equine Sport Medicine Center, School of Veterinary Medicine, University of Córdoba, 14004 Córdoba, Spain; 2 Department of Artistic and Body Education, University of Córdoba, 14004 Córdoba, Spain; 3 Department of Cellular Biology, Physiology and Immunology, School of Veterinary Medicine, University of Córdoba, 14004 Córdoba, Spain; 4 Department of Animal Medicine and Surgery, School of Veterinary Medicine, University of Córdoba, 14004 Córdoba, Spain; 5 Veterinary Teaching Hospital, School of Veterinary Medicine, University of Córdoba, 14004 Córdoba, Spain

**Keywords:** exercise, horse, rehabilitation, training, water treadmill

## Abstract

**Simple Summary:**

We know a horse has accelerometric and locomotion changes when exercising on a water treadmill (WT) associated with a better performance in dressage and jump competitions. We do not know if these changes are being maintained during terrestrial locomotion on a training track. Therefore, to answer this question, we compared locomotion on a training track before and after WT exercises at different water levels. We have found that the locomotion changes that occur during WT exercise are being maintained during terrestrial locomotion. We have also found some accelerometric evidence indicating that horses with a low fitness level can fatigue after WT, if the water was at a stifle level. This information becomes particularly important when designing a training or rehabilitation program for horses with a low fitness level.

**Abstract:**

During a water treadmill (WT) exercise, horses change their accelerometric patterns. We aimed to analyze if these changes persist during terrestrial locomotion. Six horses were randomly subjected to 40 min duration WT exercises, without water (WW), at the depth of fetlock (FET), carpus (CAR) and stifle (STF), with a day off between them. Before and after 30 min after WT, horses were evaluated at walk and at trot on a track with a triaxial accelerometer fixed on the pectoral (PECT) and sacrum (SML) regions. The percent of change from baseline (before WT and after each exercise session) were calculated. Total, dorsoventral, longitudinal and mediolateral accelerometric activities and dorsoventral displacement increased with the accelerometer in PECT but decreased after WT at STF. Velocity increased with the accelerometer in PECT but decreased with the accelerometer in SML, particularly after WT at STF. A reduction in stride frequency was found with the accelerometer in PECT. SL increased with the accelerometer in SML but decreased with WT at STF. Some accelerometric changes that happened on WT remained shortly in terrestrial locomotion. The reduction in some parameters after WT at STF depth seems to indicate fatigue. This should be considered in training or rehabilitation programs for unfit animals.

## 1. Introduction

Exercise in water is being increasingly used in sport horses, either for the rehabilitation of musculoskeletal injuries or within a training program as non-specific training exercises [1,2,3,4]. Despite many clinicians and trainers recommending its use, there are few scientific studies that describe the longitudinal kinematic and kinetic changes through rehabilitation programs for specific musculoskeletal injuries. Likewise, there are no investigations that establish validated treatment protocols. To the best of the authors’ knowledge, currently there is only one investigation assessing biomechanical changes after rehabilitation on a water treadmill (WT) vs. a treadmill without water or land treadmill [5]. After induction of osteoarthritis in the midcarpal joint, it was found that horses that rehabilitated on a WT with the water at the level of the shoulder showed a greater overall improvement in forelimb function, joint range of motion and synovial membrane integrity compared to rehabilitation without water. More recently, Potenza et al. [6] demonstrated that, after arthroscopic surgery for osteochondral fragments of the metacarpophalangeal, metatarsophalangeal or carpal joints, the rehabilitation of racehorses with a WT exercise needed less time to recover and return to racing, as the number of horses that raced after surgery was greater compared to conventional rehabilitation.

There is a variety of published studies concerning equine kinematics during exercise on a WT at different water depths [7,8,9]. Recently, we have reported the accelerometric changes that happen during an exercise session on a WT at different water depths and velocities, 5 and 6 km/h, using a validated accelerometer [2]. We found that total accelerometric activity (TAA), measured at two different anatomical locations, in the pectoral region (PECT), near to the sternum, and in the sacrum midline (SML), when representing the total acceleration patterns at both locations, achieved the greatest values with the water at the carpus (CAR) level compared to the levels of the stifle (STF), fetlock (FET) and without water (WW). The greater TAA values with the water at the level of CAR and STF would represent a benefit for some sport horses, implying that they are able to perform a greater musculoskeletal effort [10,11]. Therefore, this preliminary result would guarantee future investigations to evaluate how these parameters would be modified in horses in which WT exercise is introduced within their training and in comparison with a routine program.

Total accelerometry activity was calculated by the accelerometer from the sum of the accelerometric activities of the three body axes, i.e., dorsoventral accelerometric activity (DVAA, up–down direction), longitudinal accelerometric activity (LAA, measured in the direction of the movement) and mediolateral accelerometric activity (MLAA, side-to-side activity). Our preceding study [2] showed that the increase in TAA during WT exercise sessions at the level of CAR and STF was mainly due to the increase in DVAA. In fact, dorsoventral displacement (DVD) and DVAA increased progressively with the depth of the water, with the lowest values found when the horses were exercised WW and the highest values with the water at the level of the CAR and STF. However, this increase in DVAA was accompanied by a decrease in LAA when this activity was expressed as a percentage of TAA. These findings were attributed to the greater range of movement, principally flexion of the distal limb’s joints, as previous research demonstrated [8], which would favor the movements in a dorsoventral direction of a horse exercised on a WT.

Collection is one of the most important factors determining success and progression of training in a dressage horse because it is impossible to properly execute complex movements without having attained good basic collection [11]. With increased collection, the vertical component of the acceleration (DVAA) increased. At the same time, the forward component of the acceleration (LAA) decreased, reflecting that TAA in these horses was used to increase DVAA instead of LAA [12]. Furthermore, TAA appears to be one factor affecting a successful jump because it establishes the ballistic flight of the center of gravity and the body rotation over the obstacle during the airborne phase [13]. These accelerometric characteristics, i.e., greater TAA and DVAA, are therefore desirable for dressage, jumping and eventing horses. Interestingly, these favorable changes in the accelerometric pattern are similar to those that happened during a WT exercise, mainly with the water at the level of CAR and STF [2].

However, we have to keep in mind that all the changes found during a WT exercise session do not have to be maintained during terrestrial locomotion. Consequently, the present study was designed to elucidate whether, after performing a WT exercise session, these accelerometric modifications persist during subsequent terrestrial locomotion. With this research, we hypothesized that: (1) TAA evaluated on a track would be greater after performing a WT exercise at the level of the CAR and STF, compared to shallower water depth or WW; (2) DVAA and DVD on a track would be greater after WT exercise at the level of CAR and STF; and (3) LAA would be lower on the track after WT exercise at these same levels. If these hypotheses are proven, we would have a scientific basis to suggest that some sport horses would benefit from the inclusion of WT exercises within their training programs.

## 2. Materials and Methods

### 2.1. Horses

Six privately owned horses, two mares and four geldings aged between 10 and 15 years (mean: 12.6 ± 1.6 years) and of different breeds (one crossbred, one Arabian, one Anglo-Arabian and three Andalusian horses), were enrolled for the study. Body weight ranged between 390 and 460 kg (mean: 414 ± 45 kg).

The horses had no history of recent health issues, poor performance or lameness. Before starting the study, a complete physical (vital signs and chest auscultation) and lameness examination, hematology (red and white blood cell counts; white blood cells populations; hemoglobin concentration; packed cell volume; platelet number) and clinical biochemistry analysis (blood ureic nitrogen, creatinine, albumin, total plasma proteins, Na, K, Cl, Ca, Mg, P, total bilirubin, GGT, LDH, ALP, CK, AST and fibrinogen) were performed. Lameness examination consisted of visual examination, palpation and flexion tests. Only healthy and sound horses were included in the study.

The fitness level of the horses was moderate according to the heart rate and blood lactate response to a short-exercise test performed on a track. This test consisted of a warming-up at walk for 3 min, followed by three workloads at trot, canter and gallop for 3 min each.

The horses were recruited after their owners accepted their inclusion in the study; all of them had similar fitness levels and were used for similar purposes (leisure). Housing and feeding of the horses were not changed during the period of study. Horses were kept in a medium-size paddock, and they walked one hour per day in a walker.

### 2.2. WT Exercise

Horses performed four different trials on the WT (Activo-Med^®^, Mechtersen, Germany) that consisted of an exercise session at four different water depths: without water (WW) and with the water at the metacarpophalangeal joint (fetlock, FET), carpus (CAR) and stifle (STF) levels. The order of each of these trials, for each horse, was randomly designed (www.random.org accessed on 9 March 2021). Exercise sessions had a total duration of 40 min, including the time needed to fill and drain the WT. The velocity of the WT was fixed at 5 km/h in all the trials. Horses were fully acclimatized to WT exercise before starting the research because they were exercised for 2 weeks on the WT before starting the study.

### 2.3. Accelerometric Device

For the accelerometric evaluations, a portable 3D gait analyzer (Equimetrix, Centaure-Metrix^®^, France), a device that integrates three orthogonal accelerometers to measure the accelerations that occur along the three body axes, was well as a data logger and a software program (Equimetrix-Centaure 3D^®^) that process the acceleration signals, was placed on the horse. This accelerometer records continuous data at a sampling rate of 100 Hz while the horse is moving.

**Figure 1:**
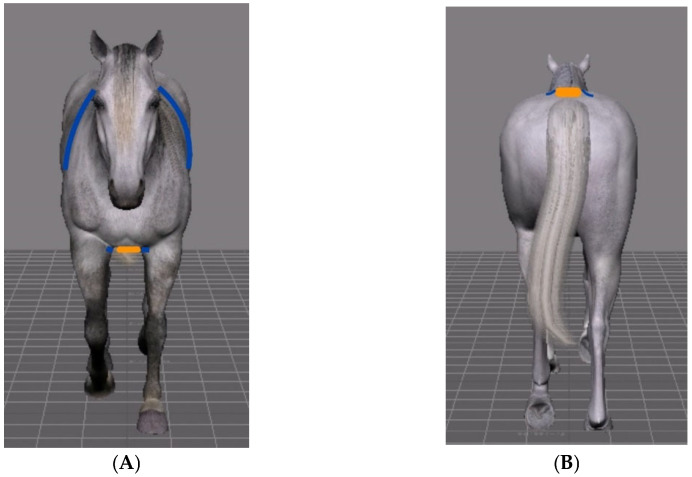
Representation of the position of the accelerometer (orange). (**A**): fixed in pectoral (PECT) position with a girth. (**B**): attached to the skin over the midline of the sacrum (SML).

The accelerometer was placed in the caudal part of the sternum (PECT) between the right and left *pectoralis ascendens* muscles at the level of the girth, as previously recommended by other authors [11,14]. This location allows the device to be near the body center of gravity, having a good stability against the body of the horse, thus providing more information about the accelerometric parameters of the forelimb. In addition, the accelerometer was attached to the skin over the midline of the sacrum (SML) using an adhesive tape to better analyze accelerometric changes in the hind limbs. A schematic representation of the position of the accelerometer is presented in Figure 1.

### 2.4. Accelerometric Evaluations

Each horse was subjected to two accelerometric evaluations in a track in each of the four trials conducted: before being exercised on the WT (baseline) and after 30 min of a WT exercise. Consequently, each horse performed four baseline accelerometric evaluations (before WT, FET, CAR and STF exercise sessions) and four accelerometric evaluations 30 min after the completion of these exercise sessions. One day off was left between each of the trials.

The accelerometric evaluations were performed with the horses at walk and at trot, led by hand and always over the same flat track surface to avoid accelerometric differences associated with the consistency of the track. Horses covered a distance of 80 m, four times for each evaluation (two at walk and two at trot). The same researcher (A.S.) positioned the accelerometer on all occasions and led the horses by hand to assure the optimal reproducibility of the recordings.

### 2.5. Accelerometric Parameters

Accelerometric activities, stride coordination and stride spatiotemporal parameters were used. Accelerometric activities included dorsoventral accelerometric activity (DVAA, W/kg), longitudinal accelerometric activity (LAA, W/kg) and mediolateral accelerometric activity (MLAA, W/kg). DVAA was calculated as the integration of the activity spectrum obtained by fast Fourier transformation (FFT) from the dorsoventral acceleration signal, which measures limb suspension and loading activity. LAA estimates the craniocaudal or longitudinal activity and was obtained as the integral of the activity spectrum obtained by FFT from the longitudinal acceleration signal. LAA measures the amount of acceleration and deceleration along the longitudinal axis. MLAA is the side-to-side activity, calculated as the integral of the activity spectrum obtained by FFT from the lateral acceleration signal. MLAA therefore measures the amount of acceleration and deceleration along the lateral axis. The sum of the three accelerometric activities (DVAA, LAA and MLAA) represents the total accelerometric activity (TAA, W/kg). Additionally, dorsoventral displacement (DVD) was calculated as an estimation of the double integration of the dorsoventral acceleration signal.

Regularity (REG, dimensionless) and symmetry (SYM, dimensionless) were considered as stride coordination parameters. REG measures the similarity of the dorsoventral acceleration patterns in successive strides in a period of time. SYM measures the similarity of the dorsoventral acceleration patterns between left and right [2,11].

Stride spatiotemporal parameters included stride frequency (SF, strides/s or Hz) and length (SL, m). The velocity was monitored with a GPS fixed to the horse.

### 2.6. Statistics

Data are presented as median and quartiles of the percent of changes from baseline values on the track for each WT exercise session. Evaluation of the normal distribution of the data was performed with a Shapiro–Wilk W test and visualization of the histograms. Data did not adjust to a normal distribution.

The differences in the percentages of variation on the track (after vs. before WT exercise) at the four depths of water (WW, FET, CAR and STF) for the two gaits (walk and trot) and with the accelerometer in two different positions (PECT and SML) were evaluated with a Kruskal–Wallis test and a Mann–Whitney test as post hoc.

The level of significance was established at *p* < 0.05. The statistical software Statistica for Windows (v.13.0) was used.

## 3. Results

### 3.1. Accelerometric Activities and Dorsoventral Displacement

**Figure 2:**
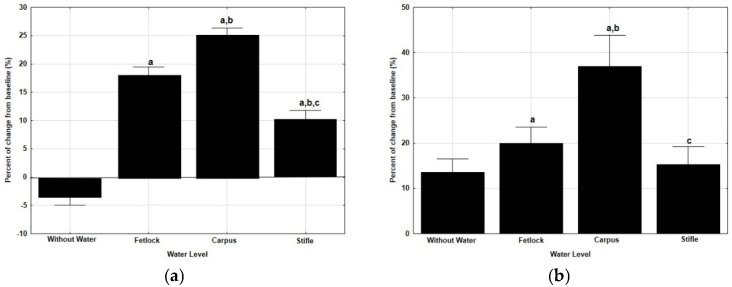

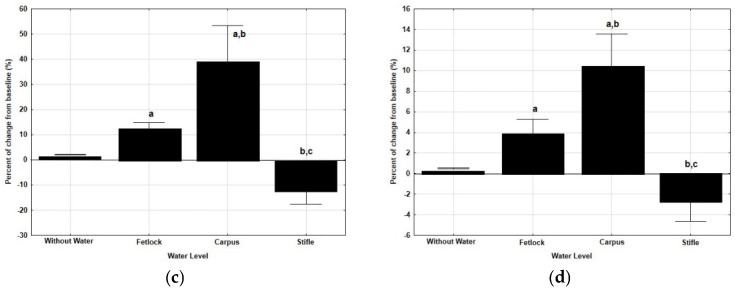
Median and quartiles of the percent of change from baseline of the total accelerometric activity, measured on a track after WT exercise sessions at four different water depths, at walk and at trot and with the accelerometer in two different positions (PECT, in the pectoral region; SML, in the sacrum midline) ((**a**): at walk with the accelerometer in PECT position; (**b**): at trot with the accelerometer in PECT position; (**c**): at walk with the accelerometer in SML position; (**d**): at trot with the accelerometer in SML position) a: significant differences with treadmill without water; b: significant differences with water at the level of the fetlock; c: significant differences with water at the level of the carpus. *p* < 0.05.

The percent of change from baseline of TAA on the track was more intense after WT exercise at the level of CAR, both at walk (*p* = 0.000) and at trot (*p* = 0.003), as presented in Figure 2. With the accelerometer in SML position, a significant reduction in TAA was found after WT exercise at STF level, at walk (*p* = 0.023) and at trot (*p* = 0.004).

**Figure 3:**
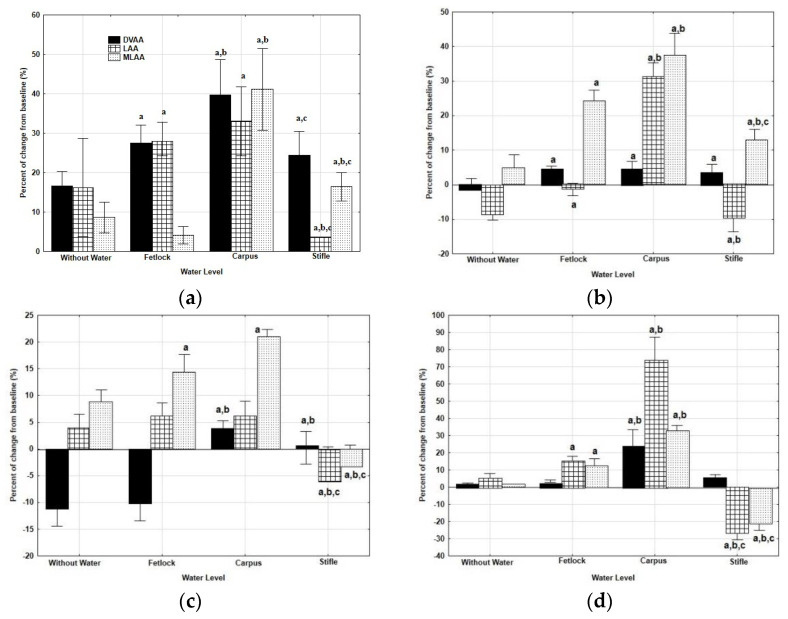
Median and quartiles of percent of changes from baseline of the dorsoventral (DVAA), longitudinal (LAA) and mediolateral (MLAA) accelerometric activities, measured on a track after WT exercise sessions at four different water depths, at walk and at trot and with the accelerometer in two different positions (PECT, in the pectoral region; SML, in the sacrum midline) ((**a**): at walk with the accelerometer in PECT position; (**b**): at trot with the accelerometer in PECT position; (**c**): at walk with the accelerometer in SML position; (**d**): at trot with the accelerometer in SML position) a: significant differences with treadmill without water; b: significant differences with water at the level of the fetlock; c: significant differences with water at the level of the carpus. *p* < 0.05.

With the accelerometer in PECT position, DVAA increased significantly on the track, showing the most marked increase after WT exercise at the level of CAR at walk (*p* = 0.004) and after WT exercise at levels of FET (*p* = 0.031) and CAR at trot (*p* = 0.028) (Figure 3). However, with the accelerometer in SML position, DVAA increased after WT exercise at CAR level (*p* = 0.032).

LAA on the track, at walk, showed a significant increase after WT exercise at the levels of FET (*p* = 0.002) and CAR (*p* = 0.000) but a decrease after WT exercise at STF level (0.001 compared to WW). With the accelerometer on SML position, LAA decreased at walk (*p* = 0.007) and at trot (*p* = 0.000) after WT exercise at STF level.

In the main lines, MLAA increased independently of gait and position of the accelerometer after WT exercise at levels of CAR (*p* = 0.000 at walk and *p* = 0.005 at trot) and FET (*p* = 0.008 at walk and *p* = 0.034). However, with the accelerometer fixed on the SML position, MLAA decreased after WT exercise at the level of STF, at walk (*p* = 0.032) and at trot (*p* = 0.004) (Figure 3).

The percent of change from baseline of DVD increased with the accelerometer in PECT position, finding the greatest increase at walk after WT exercise at the level of CAR. On the contrary, this parameter decreased at walk after WT exercise at the level of CAR and at the trot, after WT exercise at the levels of CAR and STF with the accelerometer in SML (Table 1).

### 3.2. Stride Coordination Parameters

The percent of change from baseline of REG and SYM are shown in Table 2 and Table 3. Regularity increased only after WT exercise at CAR level with the accelerometer in PECT position and at walk. Significant reductions were found with the accelerometer in SML position, at walk after WT exercise at STF level and at trot after WT exercise at CAR and STF levels (Table 2).

Stride SYM remained significantly similar after WT at the different water depths with the accelerometer in PECT position. However, it was reduced after WT exercise at the level of the STF with the accelerometer in SML position, at walk and at trot. In addition, SYM was also significantly lower at trot after WT exercise at the level of CAR with the accelerometer in SML position.

### 3.3. Stride Spatiotemporal Parameters

Very mild significant changes were found in velocity after WT exercise. It increased at walk after WT exercise at the level of CAR (*p* = 0.021) and STF (*p* = 0.033), as well as at trot after WT exercise at CAR (*p* = 0.018), but decreased at trot after WT exercise at the level of STF (*p* = 0.019). In the same way, the velocity was reduced after WT exercise at the level of STF, both at walk (*p* = 0.022) and at trot (*p* = 0.039), with the accelerometer in SML position.

Significant changes in SF were found only with the accelerometer in PECT position. At a walk, a significant increase was observed after WT exercise at the levels of FET (*p* = 0.000) and CAR (*p* = 0.005), whereas at trot, SF decreased after WT exercise at the level of CAR (*p* = 0.028).

**Figure 4:**
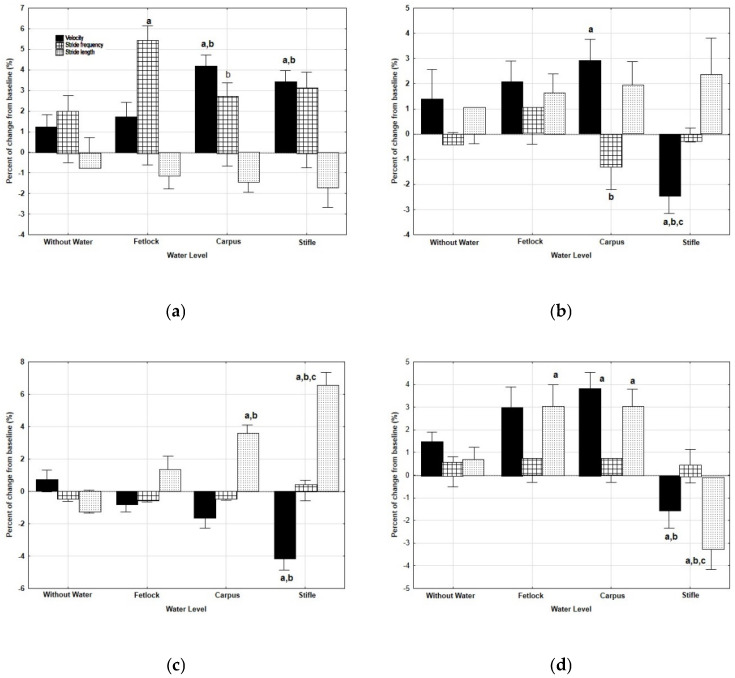
Median and quartiles of the percent of change from baseline of the velocity, stride frequency and length, measured on a track after WT exercise sessions at four different water depths, at walk and at trot and with the accelerometer in two different positions (PECT, in the pectoral region; SML, in the sacrum midline). ((**a**): at walk with the accelerometer in PECT position; (**b**): at trot with the accelerometer in PECT position; (**c**): at walk with the accelerometer in SML position; (**d**): at trot with the accelerometer in SML position) a: significant differences with treadmill without water; b: significant differences with water at the level of the fetlock; c: significant differences with water at the level of the carpus. *p* < 0.05.

With regard to SL, an increase at walk, with the accelerometer in SML position, after WT exercise at the level of CAR (*p* = 0.009) and STF (*p* = 0.000) was found. On the contrary, at trot and with the accelerometer in SML position, the percent of change from baseline was significantly reduced after WT exercise at the level of STF (*p* = 0.000) (Figure 4).

## 4. Discussion

Exercise on a WT has become popular as a training and rehabilitation tool for sport horses worldwide. This widespread use contrasts with the lack of scientific studies that support its use and lack of standardized protocols according to the objectives pursued. As cited by Tranquille et al. [1], there is little knowledge of optimal WT use and, in some cases, this could raise concerns amongst clinicians and trainers about injury development or exacerbation following its use.

In a previous investigation carried out by our research group, we evaluated the accelerometric modification in horses during an exercise on a WT at four different water depths, using the same accelerometer as in the present work [2]. The accelerometric modifications found, particularly at the CAR and STF levels, are in accordance to what has been previously described by other authors as beneficial for dressage and jumping horses [10,11,12]. This article represents the next step, with the purpose to acknowledge how the accelerometric changes that occur during a WT exercise session could affect terrestrial locomotion.

Our first hypothesis was that TAA evaluated on a track would be greater after performing a WT exercise at the level of CAR and STF, compared to shallower water or WW. This first hypothesis was only partly confirmed. Total accelerometric activity was higher on the track after WT exercise at the CAR level, with the accelerometer in both positions. However, TAA was not greater after WT exercise at the STF level, with the exception of trot with the accelerometer in PECT, which showed an increase from baseline but a decrease from the FET and CAR levels. This was an unexpected finding based on our previous data. To check that, we evaluated the individual data of TAA with the accelerometer in SML and we found that it was a fairly consistent result in the six studied horses. A plausible explanation could be fatigue as a consequence of the WT exercise at the STF level, evident in the hindquarters (accelerometer in SML position) but not in the forelimbs (accelerometer in PECT position). Despite that WT exercise at the STF level implies a greater buoyancy, the deeper water would also have increased drag force. In fact, Nankervis et al. [15] highlighted that walking on water makes a comfortable walk velocity approximately 50% lower than walking on a land treadmill or overground as a consequence of the drag force.

From these results, we might draw two practical applications. First, these results can help with tailoring an appropriate rehabilitation protocol for injured horses with low fitness levels if they have not been exercised because of injury, since the exercise on a WT at the STF level could be fatiguing. The horses included in the current investigation had a moderate fitness level. It would be interesting to see if these results can be reproduced in fitter animals. Second, a deep water level (CAR and STF) might be more helpful in improving sport horse muscle strength, even though TAA during a WT exercise was not significantly different between the CAR and STF levels in our previous research [2]. The survey performed by Tranquille et al. [1] concerning the use of WT revealed that 60% of the responders used the WT for training, mainly for dressage horses, followed by eventers and show jumpers. The main reasons for using the WT were the impression of increased strength and fitness, as well as an improvement in performance perceived by the owner or rider. It is tempting to speculate that these subjective perceptions could be linked to the higher accelerometric activities during WT exercise. Curiously, the owners/trainers in the survey of Tranquille et al. [1] indicated that deeper water (CAR/STF levels and above) was used more frequently for training compared to rehabilitation.

Our second hypothesis, based on the DVAA and DVD on the track, that it would be greater after WT exercise at the levels of CAR and STF, has been confirmed. The depth of water that induced the most evident changes in DVAA on the track was the CAR level and particularly at walk with the accelerometer in PECT position and at trot with the accelerometer in SML position. This could be related to the locomotion on the WT. At the CAR level, the horses tried to ‘step on’ the water. This movement did not appear at the STF level. Similarly, DVD increased with the accelerometer in PECT position, detecting the most marked increase after WT exercise at CAR level. Surprisingly, this parameter decreased with the accelerometer in SML position, with the greatest reductions after WT exercise at the CAR and STF levels, a result that might be interpreted as a certain degree of fatigue.

In our previous accelerometric study on WT [2], it was found that the increase in DVAA was accompanied by a reduction in LAA. Our third hypothesis was that LAA would be lower on the track after WT exercise at the CAR and STF levels. By contrast, LAA increased after a WT exercise, with the largest increase found after the CAR level. However, a reduction in LAA was found on the track at trot, with the accelerometer in PECT position and at the walk and trot with the accelerometer in SML position. It appears that these data are other indications that exercise on a WT with water at the STF level can be fatiguing despite the buoyancy effect. The MLAA, considered as an indirect indicator of the degree of flexibility [16], also increased on the track after WT exercise, detecting the greatest increase after CAR level, but showing a reduction after WT exercise at the level of STF, at walk and at trot.

To sum up with the three proposed hypotheses, with this research, we would like also to have an insight into the evolution of other stride parameters after WT exercise. Regularity and SYM were either unchanged or reduced, with the strongest reductions with the accelerometer in SML position. A significant increase in stride REG after WT exercise at CAR depth was found only at walk with the accelerometer in PECT position. Considering that REG and SYM are essential parameters for dressage horses as high values have been linked to greater scores in competition [12], these data are not favorable. The reason for these results is unknown, with the exception of considering the effect of fatigue. However, in a previous study, we found that both parameters changed very little during a WT exercise, regardless of water depth [2].

Taking into account the velocity on the track, because the horses were led by hand, the results are partly subjected to the velocity of the handler, even though the horses could freely choose velocity of movement. According to our experience, there are variations in velocity when the horses are hand-carried by different handlers, particularly at trot. However, when they are hand-carried by the same experienced and trained handler, the velocity is quite constant, without significant differences between runs.

The variation of SL and SF have been less marked than those found in the accelerometric parameters and probably they were affected by the changes in velocity. Contrary to what happened during a WT exercise, where a reduction in SF has been reported [2,7,8], in the current investigation, an increase in SF was observed on the track, with the accelerometer in PECT position and at walk. The maximum SF percent of change was observed after WT exercise at FET level, while during a WT exercise, the lowest value has been described with the water at the level of the CAR [7]. In addition, a significant reduction in SF was found at trot after WT exercise at CAR level. Regarding SL, a reduction at walk, but an increase at trot, was observed with the accelerometer in PECT position. With the accelerometer in SML position, an elongation of the stride was found at the walk and trot, after WT exercise at all water depths, with the exception of the trot after WT exercise at STF level.

Our research has some limitations. First, accelerometric adaptations have been analyzed shortly after a WT exercise session (30 min). It would be interesting to evaluate how long these accelerometric changes persist in terrestrial locomotion after a WT exercise, as well as their long-term effects on overground locomotion. Second, horses with moderate fitness level were studied. We do not know to what extent this data can be extrapolated to horses with a better fitness level. However, horses in rehabilitation can experience a reduction in fitness and, therefore, our study with moderately fit horses has importance in this sense. Third, the objective of this research was not to analyze how the inclusion of a WT exercise could affect adaptations to training or performance on competition because it will require a different experimental design, although we hope to perform this analysis in the near future.

In our opinion, the most relevant finding of our study is that, according to the results derived from the accelerometric parameters, WT exercise at the STF level should be done with caution in unfit horses, since it could lead to fatigue where velocity during the WT exercise session at this depth would need to be reduced to avoid this fatigue. The determination of the intensity of fatigue during a WT exercise is challenging. The main markers of exercise intensity used during terrestrial locomotion are heart rate response and blood lactate accumulation [17,18,19]. The heart rate rises to a lesser degree during an exercise on a WT compared to an exercise at the same velocity overground or on a land treadmill [7,20,21,22] because of the influence of buoyancy and hydrostatic pressure. In humans, it has been demonstrated that during water immersion hydrostatic pressure causes blood to move from the periphery to the thorax, increasing stroke volume and cardiac output. Consequently, heart rate remains the same or increases less than expected [23], and the same has been proposed to happen in horses [21,22]. On the other hand, it is widely documented that WT exercise is of an aerobic nature, with low blood lactate accumulation [24].

## 5. Conclusions

In conclusion, when an exercise session is performed on a WT, some of the modifications in the accelerometric pattern are maintained, at least shortly, during terrestrial locomotion. On the other hand, possible manifestations of fatigue, such as reduction in total and longitudinal accelerometric activities, dorsoventral displacement and velocity, mainly at trot and particularly with the accelerometer in SML position, have been observed when the animals were previously exercised on the WT at the STF level. We have attributed these results to a greater drag force at this depth, which would counteract the action of buoyancy.

## Figures and Tables

**Table 1:** Median and quartiles of the percent of change from baseline of the dorsoventral displacement measured on a track after WT exercise at four different water depths, at walk and at trot and with the accelerometer in two different positions. WW: treadmill without water; FET: water at the level of the fetlock; CAR: water at the level of the carpus; STF: water at the level of the stifle. 1 Position of the accelerometer: pectoral position (PECT) and sacrum midline position (SML). a: significant differences with WW; b: significant differences with FET; c: significant differences with CAR. *p* < 0.05.

WW	FET	*p*	CAR	*p*	STF	*p*
Accelerometer in PECT *^1^* position at walk						
7.17 (4.91)(10.43)	8.77 (5.00)(11.10)		16.98 (13.3)(20.21)	0.020 a 0.010 b	4.52 (2.23)(6.81)	0.030 b 0.010 c
Accelerometer in PECT *^1^* position at trot						
1.00 (−0.72)(2.72)	6.03 (4.39)(7.67)		7.860 (6.20)(9.52)		3.23 (1.53)(4.94)	
Accelerometer in SML *^1^* position at walk						
0.23 (−1.77)(2.23)	4.09 (−5.13)(13.39)	0.020 a	−10.04 (−6.61)(−13.47)	0.010 a 0.030 b	−11.58 (−8.54)(−14.61)	0.020 a 0.040 b
Accelerometer in SML *^1^* position at trot						
0.78 (−0.49)(2.07)	0.78 (−0.49)(2.07)		−0.25 (−1.64)(1.13)		−1.88 (−0.25)(−2.51)	0.030 a 0.020 b

**Table 2:** Median and quartiles of the percent of change from baseline of the stride regularity, measured on a track, at four different water depths, at walk and at trot and with the accelerometer fixed in two different positions. WW: treadmill without water; FET: water at the level of the fetlock; CAR: water at the level of the carpus; STF: water at the level of the stifle. ^1^ Position of the accelerometer: pectoral position (PECT) and sacrum midline position (SML). a: significant differences with WW; b: significant differences with FET; c: significant differences with CAR. *p* < 0.05.

WW	FET	*p*	CAR	*p*	STF	*p*
Accelerometer in PECT *^1^* position at walk						
2.64 (−5.07)(6.57)	4.56 (−5.07)(6.57)		10.92 (5.65)(14.56)	0.020 a	1.25 (5.46)(11.34)	
Accelerometer in PECT *^1^* position at trot						
−0.67 (−3.09)(4.45)	−0.43 (−3.48)(5.61)		−1.42 (−4.68)(4.59)		−1.09 (−5.52)(6.03)	
Accelerometer in SML *^1^* position at walk						
0.00 (−5.91)(6.92)	−1.97 (−5.64)(3.40)		−3.23 (−6.57)(2.39)		−6.07 (−14.39)(1.23)	0.020 a
Accelerometer in SML *^1^* position at trot						
−1.85 (−9.99)(4.40)	−4.49 (−9.22)(4.54)		−11.01 (−15.63)(−1.32)	0.020 a 0.010 b	−24.50 (−29.02)(−18.34)	0.002 a 0.010 b 0.030 c

**Table 3:** Median and quartiles of the percent of change from baseline of the stride symmetry, measured on a track, at four different water depths, at walk and at trot and with the accelerometer fixed in two different positions. WW: treadmill without water; FET: water at the level of the fetlock; CAR: water at the level of the carpus; STF: water at the level of the stifle. ^1^ Position of the accelerometer: pectoral position (PECT) and sacrum midline position (SML). a: significant differences with WW; b: significant differences with FET; c: significant differences with CAR. *p* < 0.05.

WW	FET	*p*	CAR	*p*	STF	*p*
Accelerometer in PECT *^1^* position at walk						
−5.59 (−10.29)(10.80)	0.48 (−11.36)(9.72)		−3.08 (−8.62)(7.84)		−7.72 (−1.35)(12.72)	
Accelerometer in PECT *^1^* position at trot						
−2.46 (−9.38)(5.13)	−2.31 (−8.47)(3.25)		−5.78 (−10.54)(9.34)		−5.67 (−11.23)(8.93)	
Accelerometer in SML *^1^* position at walk						
−1.55 (−5.62)(4.45)	−4.58 (−6.73)(−2.34)		−6.70 (−11.32)(−3.45)		−12.38 (−24.5)(−15.46)	0.000 a 0.020 b 0.010 c
Accelerometer in SML *^1^* position at trot						
0.00 (−7.35)(9.61)	−4.50 (−11.46)(7.51)		−18.48 (−22.45)(−8.37)	0.003 a	−29.82 (−35.21)(−23.71)	0.000 a 0.010 b 0.020 c

## Data Availability

The data that support the findings of this study are available from the corresponding author upon reasonable request.
